# HIV testing preferences in Tanzania: a qualitative exploration of the importance of confidentiality, accessibility, and quality of service

**DOI:** 10.1186/1471-2458-14-838

**Published:** 2014-08-12

**Authors:** Bernard Njau, Jan Ostermann, Derek Brown, Axel Mühlbacher, Elizabeth Reddy, Nathan Thielman

**Affiliations:** Kilimanjaro Christian Medical Centre, Moshi, United Republic of Tanzania; Duke Global Health Institute, Duke University, Durham, USA; Brown School, Washington University in St, Louis, St. Louis, USA; Hochschule Neubrandenburg, Stiftungsinstitut Gesundheitsökonomie und Medizinmanagement (IGM), Neubrandenburg, Germany; Division of Infectious Diseases, Duke University Medical Center, Durham, USA

## Abstract

**Background:**

HIV counseling and testing (HCT), an effective preventive strategy and an entry point for care, remains under-utilized in Tanzania. Limited uptake of HCT, despite the widespread availability of varied testing options, suggests that existing options may not align well with population preferences for testing.

**Methods:**

Between October and December 2011, we conducted an exploratory study in the Kilimanjaro Region to develop a conceptual framework for understanding which characteristics of HIV testing are associated with preferences for testing. Forty individuals (55% women, 53% never having tested) participated in in-depth interviews and focus groups to identify factors that influence whether and where people test for HIV.

**Results:**

A variety of discrete characteristics of testing venues, test providers, and testing procedures (e.g. distance to testing, counselor experience, type of HIV test, and availability of antiretroviral therapy) mapped conceptually to three domains: confidentiality of testing and test results, quality of HCT, and accessibility and availability of ancillary services. We noted heterogeneous preferences and demonstrate that while some test characteristics overlap and reinforce across multiple domains, others demand clients to make trade-offs between domains.

**Conclusion:**

Testing decisions appear to be influenced by an array of often inter-linked factors across multiple domains, including quality, confidentiality, and accessibility; perceptions of these factors varied greatly across participants and across available testing options. HCT interventions that jointly target barriers spanning the three domains have the potential to increase uptake of HIV testing and deserve further exploration.

## Background

In Tanzania, an estimated 1.5 million people are living with HIV, 83,000 people are newly infected each year, and with an estimated 80,000 deaths annually, AIDS continues to be a leading cause of death among Tanzanians [1, 2]. Despite the widespread availability of varied options for HIV testing, including more than 2,000 HIV counseling and testing (HCT) sites [3], and a high-profile nationwide HIV testing campaign, one third of women and half of men aged 15–49 have never tested for HIV [4]. Further, only 30% of women and 25% of men tested and received the results in the past year [1, 4].

A large body of literature describes diverse approaches to HIV testing in sub-Saharan Africa, including Tanzania [5–13]. Facility-based approaches are most common in Tanzania and include testing in standalone HIV testing facilities as well as in clinical settings. The latter includes client-initiated counseling and testing (CITC) [14] and provider-initiated counseling and testing (PITC), including Prevention of Mother to Child HIV transmission (PMTCT) services [15–17]. Other models, including home-based counseling and testing, mobile or outreach testing, which offers testing through mobile vans or organized testing events, and testing at workplaces or in schools, have also been occasionally implemented at local levels. Following considerable investment through the President’s Emergency Plan for AIDS Relief (PEPFAR) and the Global Fund to Fight AIDS, Tuberculosis and Malaria, HIV testing became available free-of-charge in Tanzania. Rapid HIV tests, now the standard of care, ensure that results are available to clients immediately after the test.

In Tanzania, voluntary counseling and testing (VCT) has been the main model through which individuals learn their HIV status. Acknowledging that client-initiated testing falls short of capturing important patient groups, the Ministry of Health and Social Welfare in 2007 developed guidelines for HCT in clinical settings, indicating that HCT should be recommended by health care providers as part of the standard of care [15, 16]. Despite this recommendation and the widespread availability of diverse HIV testing options, testing rates remain low [18–20].

Many factors contribute to limited uptake of HIV testing in Tanzania and elsewhere in sub-Saharan Africa (SSA), including psychological, cultural, economic, and other factors such as fear and stigma [21–24]. Some of these factors may be mitigated over time through community-based interventions and cultural shifts. Other barriers may be addressed, or compensated for, by making HIV testing options more attractive, more convenient, or otherwise better aligned with population preferences for testing. Different approaches have been developed to address barriers to testing, such as mobile, school, workplace, or home-based testing, couples testing, and self-testing [5, 6, 8–10]. While these approaches were often found to be effective in getting additional people to test, it is not clear which characteristics of testing options most influence individuals’ testing decisions. A better understanding of HIV testing preferences may allow for the design of testing options that better match the preferences of diverse populations.

In preparation for a structured, population-based assessment of HIV testing preferences, we conducted in-depth interviews (IDIs) and focus group discussions (FGDs) in Northern Tanzania to identify characteristics of HIV testing options associated with individuals’ preferences for HIV testing.

## Methods

### Overview

IDIs and FGDs were used to identify preference-relevant characteristics of HIV testing options, and to derive directional hypotheses with respect to their expected influence on testing decisions. First, IDIs with diverse community members were used to inform the development of FGD guides. FGDs were subsequently conducted with male and female adults who had previously tested for HIV and with others who had never tested.

Implementation, analysis, and interpretation of this qualitative study are consistent with Biomed Central’s *Relevance, Appropriateness, Transparency, and Soundness (RATS)* guidelines for qualitative research [24]: The study addresses the highly relevant research question of which characteristics of HIV testing options are associated with individuals’ preferences for testing; IDIs and FGDs with participants representing diverse experiences and opinions were considered the most appropriate methods to obtain the necessary information; the study transparently describes the sampling and analytic methods as well as ethical considerations; and the results of sound analysis and inductive identification of themes, supported by illustrative quotes, are presented in the context of the existing literature on barriers to HIV testing.

### Study setting

The study was conducted between October and December 2011 in Moshi, Tanzania. In 2012, the town had a night-time population of 184,292 [25]. At the time of the study, 18 facilities provided HCT services in Moshi, including hospitals, health centers, and free-standing VCT facilities. Intermittently, mobile and outreach testing has also been available at venues such as schools, markets, or workplaces; a prominent example was a high-profile nationwide HIV testing campaign which in 2007 and 2008 attracted more than 3 million testers, including more than 24,000 testers in Moshi [26]. For clients who test HIV positive, 8 HIV care and treatment centers (CTCs) provide access to antiretroviral therapy; an additional 13 CTCs operate in the two surrounding districts [27].

### Study participants

Purposive sampling [28] was used to recruit 4 male and 4 female IDI participants from diverse settings, including a bus stop, a market, a home, an office setting, and a guest house. Subsequently, participants in 4 FGDs, stratified by gender and HIV testing status (previously tested for HIV vs. never tested for HIV), were recruited through door-to-door contact in one of Moshi’s most densely populated wards. Twelve individuals were invited to each group. In total, 32 persons participated in FGDs; with 6 to 9 participants per group. The sampling approach was chosen to ensure the inclusion of a variety of viewpoints and diverse experiences among participants.

### In-depth interviews and focus group discussions

IDIs and FGD were conducted in Kiswahili, the official language of Tanzania. Separate interview guides were developed for IDIs and FGDs. First, semi-structured IDIs, conducted at the respective enrollment venues and lasting approximately 1 hour each, assessed motivators and barriers to HIV testing, and experiences with and attitudes toward diverse testing options. The results were used to inform the development of a FGD guide.

Next, FGDs, conducted at a health facility in the vicinity of participants’ homes, sought to identify characteristics of HIV testing options that are associated either positively or negatively with preferences for testing, and as such function as either motivators or barriers. Extant literature and results of IDIs formed the basis for a list of characteristics of HIV testing options; all HIV testing characteristics identified as potentially preference-relevant were probed and the list was iteratively expanded during consecutive FGDs. FGD participants were asked to identify factors that influence whether and where people test for HIV, and a variety of testing options, including different types of testing facilities (hospital- or health center-based testing, free-standing VCT facilities) and venue-based testing options (mobile VCT, home-based VCT, self-testing) were discussed to explore positive and negative features. Each characteristic was discussed until an understanding was developed of the mechanisms through which they influence testing decisions, and the direction of the effect could be inferred. Each FGD lasted approximately 2.5 hours.

### Data management and analysis

IDIs and FGDs were analyzed separately. IDIs were tape-recorded, transcribed, and translated into English. During the FGDs, notes were taken by two experienced recorders and two or more investigators, and expanded immediately after the discussions. Translated transcripts and text notes were read independently by multiple investigators, and a note based approach [28, 29] was used to identify characteristics of HIV testing options associated with testing preferences and testing decisions. Conceptually related characteristics were later grouped into domains. Representative, verbatim quotes from in-depth interviews and focus groups were selected to illustrate key findings.

### Human subjects considerations

The study protocol received ethical clearance from the Institutional Review Board of Duke University, the Kilimanjaro Christian Medical University College Research Ethics Committee, and Tanzania’s National Institute for Medical Research. Written informed consent was obtained from all participants. Participants were assigned numbers to ensure anonymity. Participants were compensated for their participation (approximately US$ 3.00).

## Results

Characteristics of study participants are summarized in Table 1.

**Table 1:** **Characteristics of study participants (N = 40)**

	Women (n = 22)		Men (n = 18)	
In-depth interviews	5	22.7%	3	16.7%
Focus group discussions	17	77.3%	15	83.3%
Mean age in years (range)	40 (18–57)		35 (19–60)	
Married (vs. not married)	9	40.9%	8	44.4%
Primary education or less (vs. secondary education or higher)	13	59.1%	6	33.3%
Previously tested for HIV (vs. never tested for HIV)	9	40.9%	10	55.6%

In IDIs and FGDs, a variety of characteristics of HIV testing options emerged as influencing HIV testing decisions. Characteristics were grouped into domains, with some characteristics found to be related to more than one domain.

### Domain 1: confidentiality of testing and test results

Irrespective of gender or HIV testing history, respondents indicated the importance of confidentiality for the HIV testing process and disclosure of test results. This was one of the strongest held views among the four groups, and there was no dissent as to its importance. Several respondents gave specific examples that highlighted consequences of breaches in confidentiality, including possible negative reactions by partners, relatives, employers, or others to a positive HIV test. A female informant who had never tested (“non-tester”) said, “*I am afraid if I am found to be HIV positive I may be chased away from my job.*”

#### a) Confidentiality concerns associated with venue

Perceptions of confidentiality were associated with characteristics of testing venues, as well as the counselors providing testing. There was a general consensus that hospital-based testing afforded greater confidentiality.

*“There is a big difference in confidentiality for HIV test results. In [large hospitals] there is confidentiality of client’s results. In free-standing HIV sites, you may get tested and within a few days you may start hearing people talking about your results.” (Male non-tester, IDI).*

Several participants indicated that the large size of a hospital provided a greater degree of anonymity, particularly in comparisons with an alternative such as home-based testing. Others mentioned that people go to hospitals for many reasons, masking hospital-based HIV testing. Participants voiced concern about lack of privacy in high-volume testing centers or mobile counseling and testing in tents. A male FGD participant who had previously tested said, *“You find that a center has so many people that during an interview by the counselor, others outside the room hear all that you are discussing.”* In the context of home-based testing, there was apprehension that home visits by HIV counselors, identified as such, would be noticed by neighbors; a female who had never tested was also concerned about immediate disclosure.

*“Most people are afraid to get tested for HIV at home, because people in most relationships are not faithful. So, if they test at home they have to disclose their HIV test results.”* (Female non-tester, FGD)

#### b) Confidentiality concerns associated with counselor characteristics

Counselor characteristics associated with confidentiality were primarily age and experience.

*“I would prefer a female counselor, older, around 50 years or above, with nice language, who has respect for her clients. Young counselors are not well experienced and may be tempted to expose the test results of their clients.”* (Female non-tester, IDI)

Some participants indicated a strong preference against a counselor they know or who resides in the same neighborhood.

*“When a counselor comes from your neighborhood it is bad because she could gossip … so I will go far for HIV testing where I know I will find people who don’t know me.”* (Male tester, FGD)

For others this was not important because a chance emains that they will meet the counselor again after their HIV test.

*“I came to test here at [health facility]. I did not know the counselor I found and I did not even know where she lives, but later she came to rent in my neighborhood. Therefore, I think there is no reason to know where a counselor comes from.”* (Male tester, FGD)

### Domain 2: quality of the counseling and testing procedure

Participants related characteristics of the testing venue and testing providers, and the type of HIV test, to perceptions of the quality of HIV counseling and to the accuracy of HIV tests.

#### a. Quality of counseling

Respondents underscored the importance of adequate, unhurried counseling prior to HIV testing.

*“It is very important for clients to receive adequate counseling before being tested for HIV. It is important for counselors to have enough time for the counseling sessions. For example, a counselor may want to stay for 30 minutes, while I would prefer 2 hours, so that I can be well counseled and then decide to get tested.” (Male tester, IDI)*

Concerns about adequate counseling time were voiced for high volume testing venues, including hospitals and mobile testing. Some participants considered facilities with an exclusive focus on HIV testing as more specialized than other settings, such as a hospital.

*“For other centers it is doubtful that they can be as specialized, because they provide other services. Free-standing sites provide the best kind of counseling, because they are only specialized in HIV testing.”* (Male tester, FGD)

Age and experience were the most commonly mentioned counselor characteristics associated with testing preferences. While many participants had no gender preference, several informants, primarily females, preferred to be tested by female testers. However, these preferences were related more to personal comfort than concerns about quality. Some participants preferred to be tested by doctors rather than nurses or HIV counselors.

*“I would prefer a doctor, because a doctor is more knowledgeable than a nurse … The doctor should be 40 years or older and experienced in HIV counseling and testing.”* (Female non-tester, FGD)

#### b. Accuracy of HIV tests at different venues

Participants believed that the accuracy of tests differed between testing sites. Reasons for such differences included the availability of more than one type of test, the use of multiple tests, and the training of those administering the test.

*“There are differences in HIV tests in free-standing sites compared with hospitals. For example, you may test at a free-standing site, and receive positive HIV results. Nevertheless, if we decide to re-test in a hospital you may get negative HIV results. In addition, at a hospital you may test urine, sputum, and saliva,* etc.” (Male non-tester, IDI).

Informants generally associated large hospitals with more accurate HIV tests; private facilities were associated with less accurate HIV tests. A male IDI informant who had never tested, said, “*Private HIV counseling and testing sites […] don’t use accurate HIV tests. I don’t trust the results from such sites.*”

Counselor training was also mentioned as a reason for differing accuracy between venues. Counselors who test many people at a large hospital were perceived to provide more accurate results. By contrast, a female tester was concerned about mobile testing:

*“It is possible that people who conduct the mobile testing are untrained and may fail to interpret my HIV results correctly. You may receive incorrect HIV test results, and this may cause unnecessary anxiety.”* (Female tester, FGD)

#### c. Accuracy of different HIV testing procedures

Significant discussion revolved around the accuracy of different HIV testing procedures. While several participants suggested that fear of needles is an important barrier to testing, when asked about their preference for specific procedures, the primary concern was accuracy. Participants were roughly evenly split between those who preferred venipuncture and those who preferred a finger prick for obtaining the blood sample. Participants who preferred venipuncture generally considered blood samples from a large vein in the arm to be more reliable than a finger prick for HIV testing.

*“Blood from a finger prick is to test for Malaria. Blood to test for HIV comes from the big vein, because it flows with pressure and I will be sure that my HIV test results will be accurate.”* (Female tester, FGD)

Self-test kits were not considered a feasible option for wide-spread HIV testing in this setting; participants voiced concerns about accuracy, and about the lack of support from trained counselors to assist with a positive test result.

*“I won’t be sure if the self-test results are accurate. For example, I won’t be sure on how long the reagents have stayed in the drug shop/or pharmacy. In such cases, I may get an incorrect test results. It will be very difficult.”* (Male tester, IDI)

Literacy concerns were also raised.

*“Most people, particularly in the rural areas are illiterate; they can’t read even a newspaper. How can they be able to read and follow the instructions of how to use the HIV testing kits?”* (Male tester, IDI)

### Domain 3: accessibility of testing and other services provided

Additional discussion centered on the importance of distance to the testing venue, transport cost, testing times, and waiting times for the accessibility of HIV testing, and on options for making testing more attractive, by offering HIV testing in conjunction with other services or even paying people to test.

#### a) Accessibility of HIV testing

Many participants did not consider transport costs a barrier in an urban setting due to the availability of local testing sites, however, others indicated that they, or other people, may prefer not to test close to home:

*“Personally, I will not go far to test for HIV. But there are people in the community who travel to other places to test for HIV.”* (Male non-tester, IDI)

Most participants indicated that they are ready to wait for a substantial amount of time before seeing a counselor. However, a longer waiting time was also associated with an increased risk of compromising confidentiality; a female tester was concerned that “*someone who knows you may come*”. (Female tester, FGD)

Time conflicts with other activities were only mentioned in the context of home-based testing.

*“[……..] it depends on my time availability. For example a health worker may come, while I am leaving to go to work, or any other activities. I will not agree to test because of lack of time."* (Male tester, IDI)

Nonetheless, participants indicated that providing HIV testing services off hours or during weekends could be beneficial to some, particularly to people who are busy or employed.

#### b) Ancillary services

Availability of antiretroviral medications at the testing site was perceived as a motivating factor for HIV testing among some respondents. A male participant preferred to test in a hospital setting as the transition from being a testing client to becoming a patient would be smoother.

*“… you receive your results on the spot and if you have any problems it is easier to see a doctor or enroll for HIV treatment or other support.”* (Male non-tester, IDI)

Other participants indicated that the availability of other services would help to reduce stigma and increase convenience.

Several female discussants mentioned antenatal care as an entry point for their first testing experience; and both male and female discussants saw antenatal care as an opportunity to get more men to test.

*“Pregnant women should be encouraged to ask their spouses or partners to accompany them to clinic. It should be conditional, that if they are not accompanied by their spouses or partner, then they will not receive any services or retain their clinic attendance cards. They will come in the next visit with their spouses or partners.”* (Female non-tester, FGD)

#### c) Payment for testing

Testing is generally provided free of charge in the study area. Respondents reacted very differently when asked if they would agree to test for HIV if they are paid. Several participants disagreed strongly with payments for testing, as illustrated by a male non-tester.

*“No. Because that will look like I am tempted to be tested for HIV and it is not my own decision or will. It will appear as if I am being bought.”* (Male non-tester, IDI)

Others suggested that payments could be used to get more people to come for testing.

*“My experience is that if it is announced that people will be paid Tsh 5,000/= (US$3) to test for HIV, and considering the harsh economic conditions, the space at [VCT site] will be small.”* (Male tester, FGD)

Payment in the form of a transport cost reimbursement was also considered to have the potential to influence testing decisions, or enable people to mitigate confidentiality concerns, as indicated by a female discussant who said, “*If my transport costs will be reimbursed, I will travel outside Moshi to get tested for HIV.*” (Female non-tester, FGD)

### Concept mapping

**Figure 1:**
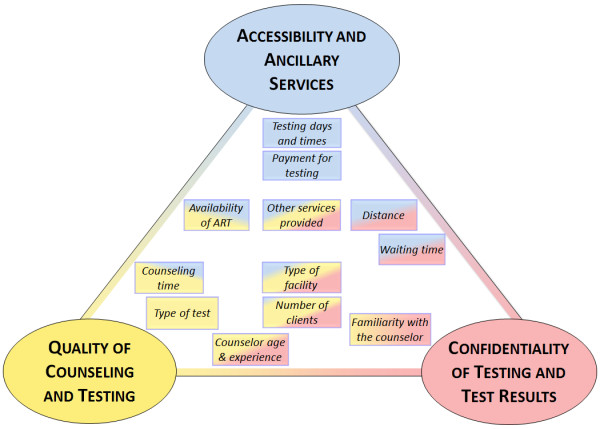
**Concept map of HIV testing characteristics.**

Figure 1 summarizes the preference-relevant characteristics of HIV testing options identified in FGDs and IDIs (in boxes) and visually describes their relationship to the three underlying domains (in ovals). The domains are broadly defined as quality of counseling and testing, confidentiality of testing and test results, and accessibility and ancillary services. The quality domain includes characteristics such as the accuracy of the HIV test, the perceived quality of testing procedures, adequate time for counseling, and the experience of counselors. The confidentiality domain includes characteristics perceived to be associated with a potential disclosure of HIV test results (e.g. by a younger counselor, or in a mobile setting) and characteristics associated with the inadvertent disclosure of testing *per se*. Examples for the latter include testing close to home, familiarity with the counselor, testing at a facility that only offers HIV testing, and long waiting times or large numbers of clients that increase the risk of familiar encounters. The accessibility domain describes the time and monetary cost of accessing testing, and opportunities for combining HIV testing with other services.

Most characteristics of HIV testing options related to multiple domains. For example, counseling by a counselor who is known to a participant may be associated with perceptions of a higher quality of counseling or with concern about confidentiality. Similarly, the type of facility is associated with the concepts of quality (e.g. perceived more accurate test results in hospitals or better counseling in dedicated HIV testing facilities), accessibility (e.g. by combining HIV testing with other screening or treatment services), and confidentiality (e.g. by having other reasons to be seen at a hospital). Associations between characteristics and domains are indicated by the placement and shading of each characteristic. The multi-faceted relationship of testing characteristics with the three domains is indicated by each box’s shading with multiple colors.

## Discussion

In-depth interviews with key informants and focus group discussions elucidated a variety of preference-relevant characteristics of HIV testing options, which map conceptually to three domains: confidentiality, quality, and accessibility.

Not surprisingly, as others have clearly documented [21–23, 30], concerns about confidentiality were preeminent and likely to affect HCT utilization. Our data highlight a level of unease about potential inadvertent disclosure of HIV status or HIV testing in association with specific testing venues, including free-standing and mobile VCT sites and testing at home. Similar apprehension was expressed about HIV testing conducted by young counselors, who were perceived to be possibly less discreet than older ones. Because the desire for confidentiality is linked to stigma, our findings highlight the importance of addressing both confidentiality and stigma in the design of newer approaches to HCT.

Concerns about accuracy were related to specific venues, the training of test providers, and the type of test. Some respondents feared that private or free-standing testing sites were prone to providing false positive results, whereas counselors with more experience, especially those working in large hospitals, are likely to deliver more accurate results. Variation in perceived accuracy was also related to differences in tests and testing procedures across venues. Home testing did not appear to be a popular option, even among those who had previously tested, in part due to confidentiality concerns, but also because the quality of home testing was potentially suspect. It has been previously documented that perceived unreliability of test results and distrust of HIV testing technologies can discourage uptake of HIV testing [22, 31, 32].

The findings highlight the conceptual overlap between testing venue, counselor, and test characteristics and suggest that individuals make important trade-offs in considering testing options. Some prefer to test at venues that see more clients, where they perceive the accuracy of the test to be better. Others, concerned more about confidentiality, may be willing to sacrifice perceived more accurate testing at a high-volume testing center for a perceived lower risk of being seen testing at testing sites with fewer clients The heterogeneity of preferences and the complex links between the domains of quality, confidentiality, and accessibility should be accounted for in the design or re-design of testing options.

To address both confidentiality and quality concerns, the integration of testing services into a hospital or health center setting may be preferable to isolated testing services offered at free-standing facilities. As attendance at free-standing HIV testing sites appears to be declining [33], plausibly because clients prefer to access HCT services within health facilities, HCT policy makers should examine ways to re-define the roles of free-standing VCT sites in this context.

The focus group discussions demonstrated heterogeneous preferences with respect to the accessibility of testing. For many participants, distance was not a significant barrier due to the local availability of a variety of testing options. For others, traveling seemed advantageous, as testing done farther from home is more likely to be confidential. Disparate views were expressed regarding direct payments as means of increasing accessibility of testing. We note that in the context of selected studies that addressed a slightly different question, high value conditional cash transfers, given in exchange for testing negative for sexually transmitted infections, were associated with reduced infection [34–36].

With the introduction of newer approaches of HCT delivery such as the use of community based lay counselors [37], couples counseling and testing [7, 8, 38], provider-initiated [39, 40], home based [5, 11, 12], and mobile HCT [13, 23, 41], it is important for planners of HIV testing interventions to recognize that many barriers are inextricably linked. Some are overlapping and reinforcing (e.g. concerns about both quality and confidentiality with home testing), and others demand that patients make trade-offs as they choose to test (e.g. paying for travel to reduce risk of disclosure within one’s community). Novel approaches to HCT delivery must weigh the benefits of addressing heterogeneous preferences against the costs and complexities of addressing the multi-faceted and interlinked barriers.

### Limitations

Our study is subject to important limitations. IDIs and FGDs afforded an opportunity to identify a variety of characteristics of HIV testing options associated with preferences, and to begin to understand which features are most important. However, our study suggests significant preference heterogeneity among participants, which precluded the development of a consensus regarding the relative importance of specific characteristic to participants, or to differentiate the preferences of individual sub-groups. The existence of heterogeneous preferences has been confirmed by a quantitative follow-up study in the area [42].

Second, it is not clear how participants’ stated preferences relate to actual testing decisions. The preferences and characteristics of individuals are likely to interact with characteristics of testing options (e.g. venue, method for obtaining the sample for the HIV test) to influence actual testing decisions. Further, due to the focus of FGDs and IDIs on characteristics of testing options, several important elements of the decision process could not be explored in detail, including differences between first-time and repeat testing, external motivators, such as social support for testing, and internal barriers to testing, such as fears of knowing the result.

Finally, an inherent weakness of this qualitative study is that the findings may not be representative of the population in the study area and may not be applicable to other settings. Because study participants were recruited from an urban setting with comparatively wide-spread access to a variety of HCT services, our findings may not be as relevant in rural areas.

## Conclusion

This study identified several important attributes of HIV testing options that are associated with HIV testing preferences. Testing decisions appear to be influenced by an array of often inter-linked factors across multiple domains, including quality, confidentiality, and accessibility; and perceptions of these factors varied greatly across participants and with available testing options. HCT interventions that jointly target barriers across these domains have the potential to increase uptake of HIV testing and deserve further exploration.
